# Tibial periosteum distraction for the treatment of lower extremity varicose vein ulcers combined with deep vein thrombosis: A case report

**DOI:** 10.1097/MD.0000000000045252

**Published:** 2025-10-17

**Authors:** Peng Chen, Yi You, Boyang Liu, Naxin Zeng, Da Zhong

**Affiliations:** a Department of Orthopedics, Xiangya Hospital, Central South University, Changsha, Hunan, China; b Department of Cardiac and Vascular Surgery, Yueyang Central Hospital, Yueyang, China.

**Keywords:** tibial periosteum distraction, varicose veins, venous leg ulcers

## Abstract

**Rationale:**

Lower extremity venous ulcers are highly prevalent in elderly populations. These chronic wounds often persist for extended periods, heal poorly, and frequently recur, severely compromising patients’ quality of life while creating considerable socioeconomic burdens. When complicated by deep vein thrombosis, they further restrict viable treatment options.

**Patient concerns:**

A 55-year-old man had experienced left lower extremity varicose veins for 3 months, accompanied by a recurrent 3 × 4 cm fascial-penetrating ulcer, chronic pain, superficial venous dilation, and cutaneous hyperpigmentation. Doppler ultrasound revealed left lower extremity deep vein thrombosis.

**Diagnoses:**

The clinical evaluation confirmed primary diagnoses of left lower limb varicose veins (Clinical–Etiology–Anatomy–Pathophysiology-6 classification) and left peripheral-type deep venous thrombosis in the lower extremity.

**Interventions:**

The first surgery involves stripping and ligation of the superficial varicose veins at the ulcer site, in conjunction with tibial periosteum distraction. After 14 days of periosteal distraction, the distraction device was surgically removed in the second procedure.

**Outcomes:**

Postoperatively, the patient experienced substantial symptom relief in the affected lower extremity. Within 1 month, the ulcer formed a scab and approached healing by one and a half months. Throughout the 9-month follow-up, there was no recurrence of the ulcers, and the patient's quality of life markedly improved.

**Lessons:**

In patients with Clinical–Etiology–Anatomy–Pathophysiology-6 varicose veins complicated by deep vein thrombosis, the combination of tibial periosteum distraction with conventional stripping and ligation of superficial varicose veins resulted in symptomatic relief, promoted ulcer healing, reduced economic burden, and improved quality of life.

## 1. Introduction

Varicose veins of the lower extremities are dilated subcutaneous varicose veins ≥ 3 mm in diameter, caused by venous valve insufficiency resulting in increased venous pressure due to blood reflux. As the condition progresses, it manifests as pain, swelling, itching, bleeding, and skin changes that increase the risk of thrombosis and ulceration.^[1]^ Lower extremity venous ulcers have a high incidence in the elderly population, and their long duration, low healing rate, and high recurrence rate have a serious impact on the quality of life of patients, as well as a heavy socio-economic burden.^[2]^ This report presents a case of a patient with lower extremity varicose vein ulcers complicated by DVT. We adopted tibial periosteal distraction (TPD) of the left lower limb, combined with stripping and ligation of the located superficial veins and artificial dermal scaffold placement treatment modality for this patient, and after postoperative observation and post-discharge follow-up, his ulcer gradually healed, and pain was relieved, which greatly improved the patient's quality of life.

## 2. Case report

**Figure 1.**
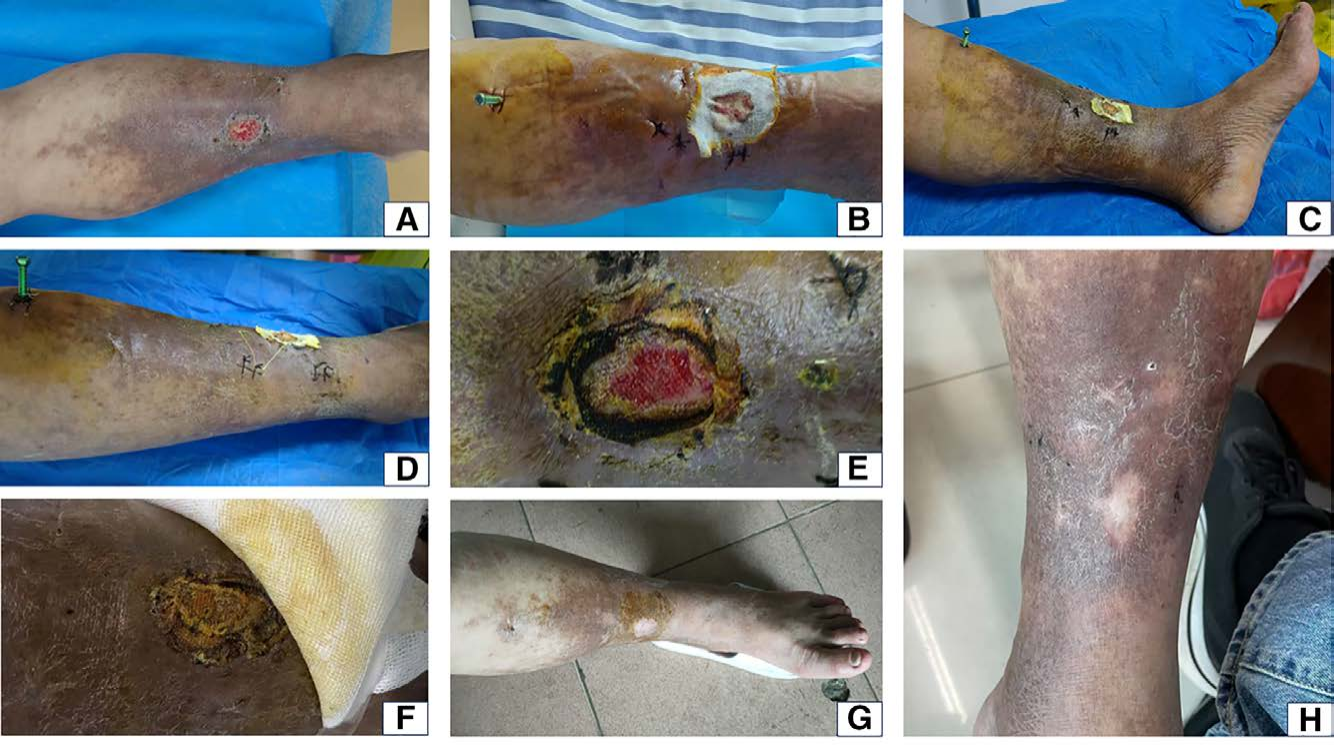
Condition of the affected limb before and after surgery. (A) Ulcer penetrating deep fascia. (B) Post-op day 2. (C) Post-op day 3. (D) Post-op day 7. (E) Post-op day 10. (F) 10 days post-periosteal device removal. (G) 15 days post-discharge. (H) 9 months post-discharge.

A 55-year-old farmer patient was admitted to our institution due to varicose veins accompanied by ulceration in the left lower extremity. The patient presents with complaints of chronic pain in the varicose vessels of the left lower extremity, persisting for 3 months, with ulceration occurring 1 month prior. No other special medical history. Upon physical examination, it was observed that the patient exhibits pronounced dilatation of the superficial veins in the lower extremities, most notably around the calf and ankle regions, accompanied by hyperpigmentation on the surface of the skin. A wound measuring 3 × 4 cm, penetrating to the deep fascia (Fig. 1A). The vascular Doppler ultrasound suggests the lower extremity deep venous thrombosis in the left lower. The absence of pain and swelling in the limb leads to the consideration that the thrombus may be old in nature. Based on clinical and examination findings, the final primary diagnoses were lower limb varicose veins (left, Clinical-Etiology-Anatomy-Pathophysiology-6) and lower extremity deep venous thrombosis (left, peripheral type).

**Figure 2.**
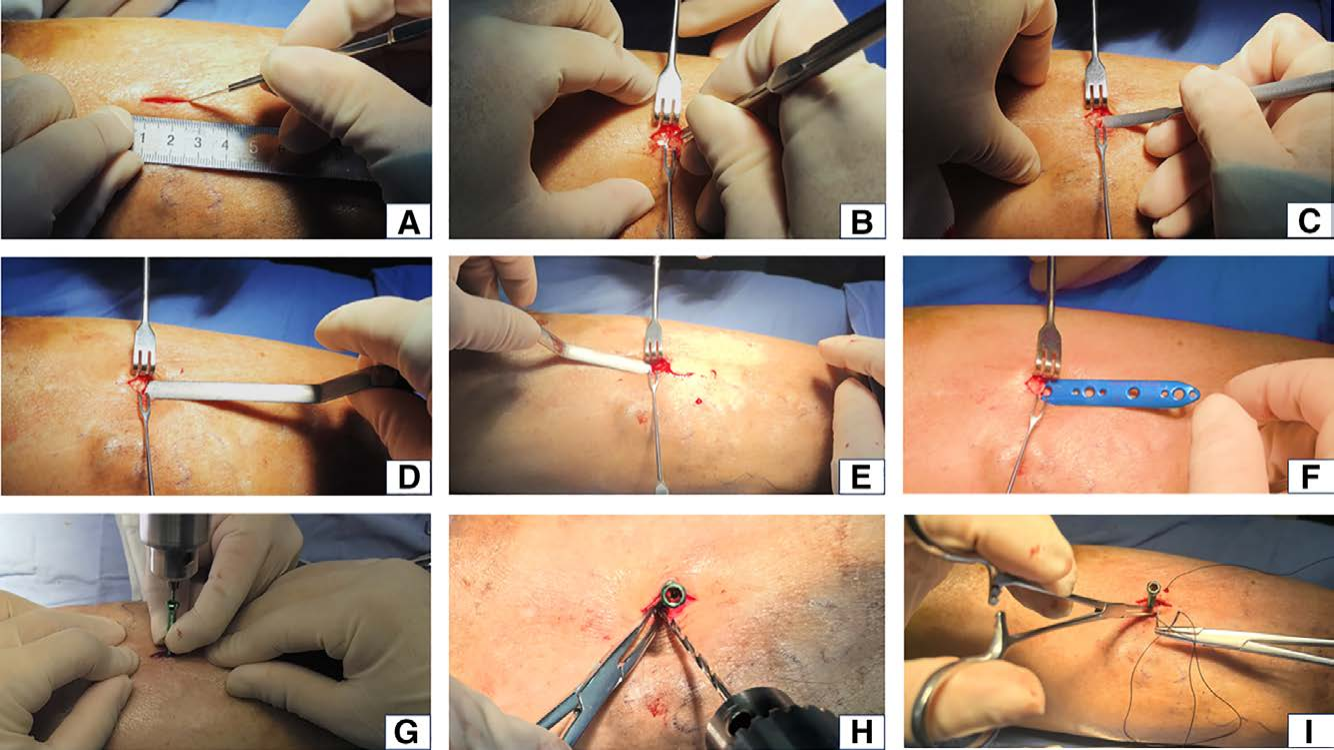
Surgical procedure for tibial periosteum distraction. (A) A 1.5 to 2.0 cm longitudinal skin incision was made. (B) Blunt dissection was performed down to the periosteal surface, followed by a 1 cm transverse incision of the periosteum. (C and D) A subperiosteal tunnel was created using a periosteal elevator. (E and F) The distraction plate was inserted, and a flat-headed hollow locking distraction screw was placed through the central hole. (G) A 2 mm Kirschner wire was drilled through the hollow screw, penetrating both cortices to secure the distraction system. (H) Cortical fenestrations were created at the 4 corners surrounding the plate to decompress the medullary cavity and induce microfractures. (I) The periosteum and skin were closed with 2 sutures each.

**Figure 3.**
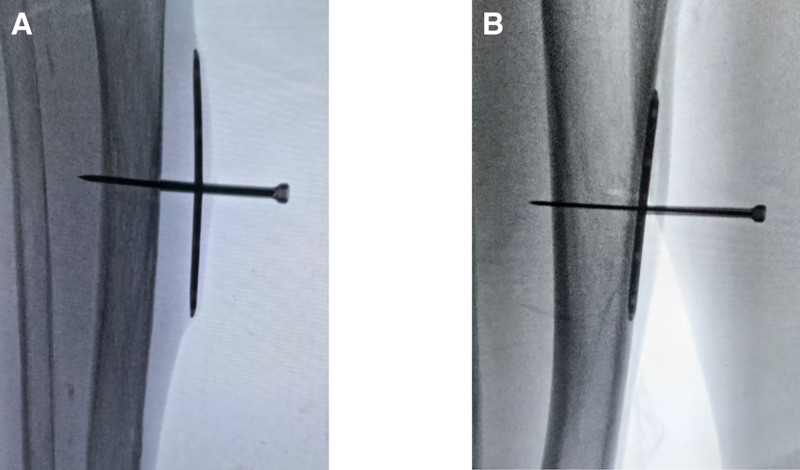
Verify the successful installation of the periosteal distractor. (A) Turn the screw clockwise to lift the steel plate with the periosteum. (B) Reverse it to reposition the plate tightly against the cortex.

We have devised a tailored treatment plan for this patient: stripping and ligation of the superficial varicose veins at the ulcer site, in conjunction with tibial periosteum distraction. The patient consented to the treatment plan. The surgical procedure is shown in Figures 2 and 3.

Starting from the third postoperative day, we gradually and uniformly increased the tension on the tibial periosteum, causing the plate underneath the periosteum to gradually separate from the cortical bone at a rate of approximately 0.75 mm/day. Patient mobility was not restricted during this period.

During the periosteal distraction period, the wound area gradually decreased in size, and the severity of left lower limb varicose veins diminished compared to previous observations (Fig. 1B–D). By postoperative day 10, fresh granulation tissue was visible (Fig. 1E). On postoperative day 17, the periosteal distraction device was removed. By the tenth day after the second surgery, the wound had formed a scab, with no tenderness upon touch, and the distal limb circulation was good (Fig. 1F). Half a month after discharge, the ulcer had virtually healed (Fig. 1G), and there was no recurrence for 9 months (Fig. 1H). The Visual Analog Scale score improved from 6 (moderate pain) preoperatively to 2 (mild pain) 9 months postoperatively. Dorsal foot skin temperature increased compared to preoperative levels. The patient indicated that the healed wound substantially enhanced quality of life, and pain alleviation permitted ambulation over longer distances. He demonstrated high satisfaction with the recovery outcomes.

## 3. Discussion

At present, the application of tibial periosteal distraction is mainly focused on the treatment of chronic ischemic lower limb diseases (e.g., diabetic foot), and it has achieved encouraging efficacy.^[3–6]^ The phenomenon of conducting distraction procedures at a site distant from gangrenous or ulcerative infections, culminating in the resolution of recalcitrant foot ailments, is referred to as the “Summon Effects.”^[7]^ Related literature compared the outcomes of periosteal distraction and tibial transverse transport (TTT) in addressing diabetic foot complications, substantiating the similarity in their clinical efficacy to that of the transverse tibial bone transport procedure.^[8,9]^ However, they concurrently noted that the periosteal distraction technique lacks the fenestration decompression of the tibial medullary cavity and the translocation of osseous tissues, which may render it less effective in promoting angiogenesis and wound healing compared to transverse tibial bone transport. Therefore, integrating existing research, we augmented the surgical procedure with a medullary cavity fenestration and drilling step, which may be beneficial in alleviating inflammation and pain in patients.

The precise mechanisms underlying periosteal distraction remain unclear. Wang et al applied TPD to the treatment of chronic limb-threatening ischemia and inferred that the acceleration of wound healing and pain alleviation by TPD may be attributable to a decrease in tibial intramedullary pressure and the mobilization of systemic stem cells.^[3]^ Before these developments, a substantial body of research has corroborated the significance of the periosteum in distraction osteogenesis.^[10–13]^ Distraction osteogenesis is contingent upon angiogenesis, with neovascularization being predominantly supplied by the periosteum.^[14]^ Research has indicated that the simple stretching of periosteal tissue on the rabbit mandible significantly promotes the proliferation of the capillary network within the distracted region.^[15]^ An increase in blood flow signifies a heightened potential for regeneration. This is corroborated by the findings of Cao et al, who demonstrated that the modified Ilizarov tibial transverse transport technique, where the fibular periosteum of the open window bone flap was retained, regenerates a more extensive microvascular network compared to conventional techniques.^[16]^

Building upon the foundational research on TTT and the existing clinical studies on tibial periosteum distraction.^[17–21]^ We hypothesize that tibial periosteal distraction is predicated upon the principles of tension and stress, engendering intramedullary hemorrhage and facilitating communication between the intramedullary and extramedullary spaces. By applying gradual and consistent traction to the periosteal tissue, this technique induces volumetric alterations within and beyond the medullary cavity, thereby stimulating tissue regeneration. The sustained, gentle traction on the periosteum fosters the regeneration of the vascular network at the extremities, enhancing peripheral blood supply, mitigating ischemic symptoms, diminishing pain, and expediting the healing process. The specific mechanisms by which these effects are mediated remain to be elucidated through further research.

Compared to TTT, we believe that TPD is a more minimally invasive procedure and has the advantage of low risk, low time, and low monetary burden. TPD is a subset of TTT, and TTT involves the distraction of a larger volume of tissue, potentially yielding superior outcomes. Therefore, the choice of surgical procedure should be balanced against the severity of the patient's condition. As a single-case report, this study has inherent limitations, such as the absence of a control group. This case report is consistent with its exploratory nature within the Idea, Development, Exploration, Assessment, and Long-term Follow-up (IDEAL) Stage 1 framework. Consequently, future well-controlled clinical trials are required to provide definitive conclusions regarding the technique's rationale and efficacy.

## Acknowledgments

We extend our heartfelt thanks to the patient who willingly participated in this study and consented to the publication of the article. The successful management of this patient benefited critically from the advice of Dr. Naxin Zeng, for which we are deeply grateful.

## Author contributions

**Conceptualization:** Naxin Zeng.

**Data curation:** Boyang Liu.

**Methodology:** Yi You.

**Supervision:** Da Zhong.

**Writing – original draft:** Peng Chen, Naxin Zeng.

**Writing – review & editing:** Da Zhong.
